# Stability of Multispecies Bacterial Communities: Signaling Networks May Stabilize Microbiomes

**DOI:** 10.1371/journal.pone.0057947

**Published:** 2013-03-04

**Authors:** Ádám Kerényi, Dóra Bihary, Vittorio Venturi, Sándor Pongor

**Affiliations:** 1 Institute of Biophysics, Biological Research Centre of the Hungarian Academy of Sciences, Szeged, Hungary; 2 Faculty of Information Technology, Pázmány Péter Catholic University, Budapest, Hungary; 3 Group of Bacteriology and Plant Bacteriology, International Centre for Genetic Engineering and Biotechnology, Trieste, Italy; 4 Group of Protein Structure and Bioinformatics, International Centre for Genetic Engineering and Biotechnology, Trieste, Italy; Semmelweis University, Hungary

## Abstract

Multispecies bacterial communities can be remarkably stable and resilient even though they consist of cells and species that compete for environmental resources. *In silico* models suggest that common signals released into the environment may help selected bacterial species cluster at common locations and that sharing of public goods (i.e. molecules produced and released for mutual benefit) can stabilize this coexistence. In contrast, unilateral eavesdropping on signals produced by a potentially invading species may protect a community by keeping invaders away from limited resources. Shared bacterial signals, such as those found in quorum sensing systems, may thus play a key role in fine tuning competition and cooperation within multi-bacterial communities. We suggest that in addition to metabolic complementarity, signaling dynamics may be important in further understanding complex bacterial communities such as the human, animal as well as plant microbiomes.

## Introduction

Members of complex bacterial communities communicate and cooperate via the exchange of public goods and chemical signaling molecules, while also compete for space and resources. Examples of such communities include microbial mats of the oceans, the gut microbiota of animals and many insects as well as the microbial communities of the rhizosphere [1]. In spite of internal competition and changing environments, multispecies communities can maintain remarkable stability over time and resiliency with respect to environmental challenges [2]. For instance, the mammalian gastrointestinal tract hosts an especially complex microbiota that is capable of resisting invasion by pathogens. For a successful colonization, incoming pathogens have to be able to scavenge nutrients, to sense community signals, to compete with the resident bacteria, and to timely regulate virulence genes [3], [4], [5]. For instance, the microflora of the human oral cavity is estimated to approximately 10^10^ bacteria belonging to about 100 different bacterial species [6], [7], [8], [9]. These species have been shown to interact via mutualistic metabolic exchanges [10], [11].

Quorum sensing (QS), a molecular regulatory mechanism in response to bacterial cell density, is used by many bacterial communities to communicate, synchronize and regulate behavior [12], [13], [14]. QS is a cell-cell communication process wherein bacteria emit diffusible autoinducer signal molecules that allow them to monitor population density, and to turn on various phenotypes in a precisely coordinated manner. Examples include secretion of exoenzymes, of siderophores (iron-chelating compounds), production of anti-microbial secondary metabolites, biofilm formation, bacterial movement, bacterial conjugation and regulation of virulence associated factors [15], [16], [17]. This synchronous response confers bacterial populations a degree of multicellularity that couples individual cell responses to population-wide alterations. The fundamental steps are comparable in virtually all QS systems [13]. In a canonical system, the autoinducer molecules are passively released or actively secreted outside of the cells. As the number of cells increases in an environment, the extracellular signal concentration likewise increases, and when it exceeds a minimal threshold level, cognate receptors bind the autoinducers and trigger signal transduction cascades that regulate gene expression. Acyl homoserine lactones (AHLs) are believed thus far to be the major class of QS autoinducer signals used by Gram-negative bacteria. These molecules have a conserved homoserine lactone ring with an acyl side chain, which may vary from three to 18 carbons. In an AHL-QS circuit, AHLs are synthesized by a LuxI-type protein, and above a critical concentration, the AHL molecule binds a LuxR-type protein. This protein is then activated by exposing a DNA binding domain that subsequently recognizes a palindromic lux box cis-element localized in the promoter region activating the expression of the target genes (8). There is also a growing list of LuxR-type proteins that function in the apo-form, and are inactivated by AHL binding [18]. Importantly, the *luxI* and *luxR* genes are often under a positive induction feedback loop forming a regulatory circuit that generates rapid amplification of the signal.

Sharing of AHL signals by QS bacteria is not uncommon in nature. Namely, AHL signals fall into closely related structural classes and it is not rare that an AHL regulator receptor can react to more than one of the chemically related signals. This phenomenon is often ascribed to the “relaxed specificity” or “promiscuity” of the LuxR regulator-receptor protein [19], [20]. The benefit that this brings to a bacterial cell is currently not well understood. Furthermore, it has been noted that some regulator-receptor proteins perceive certain signals at very low concentrations while others at much higher concentrations [21].

While there is a substantial amount of theoretical and practical work on the stability of biological communities, relatively little is known about the stability of quorum sensing communities. Recently it was shown by experiment that a wild type *Pseudomonas aeruginosa* can form stable binary communities with its mutant that is defective in signal production but able to contribute to the public goods [22]. On the other hand, a non-cooperating mutant that does not contribute to public goods can invade and collapse a wild type community. The same study also showed that the existence of QS signaling is sufficient to reproduce the above behavior patterns *in silico* (see details in Methods).

In certain diseases, small cohorts of bacterial species appear to mediate disease progression and that they do this via mutually understanding each other’s signals, a process now referred to as interspecies signaling [23], [24]. For instance, in the olive-knot disease of the olive tree (*Olea europaea*), the causative agent is the bacterium *Pseudomonas savastanoi pv. savastanoi* (PSV). However, two otherwise non-pathogenic bacterial species, namely *Pantoea agglomerans* (PA) [25] and *Erwinia toletana* (ET) [26], are often found associated with the olive-knot and the three species in the knot grow more together than alone [27]. As the three species are stably associated and appear to increase the fitness of each other, there is reason to believe that these associations are not coincidental. Recently it was shown that PSV, ET and PA not only form stable communities but also react to the signals of one another [27]. As a result we became interested in the potential role of signaling in the stability of multispecies QS communities.

**Figure 1:**
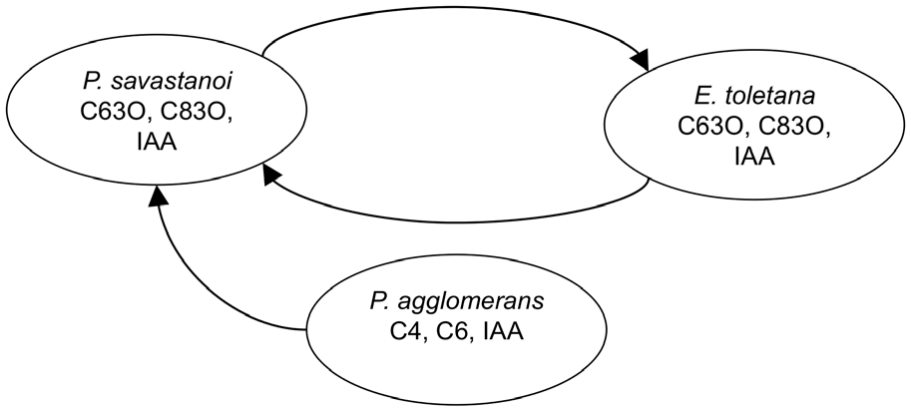
Experimentally observed sharing of bacterial signals and public goods in olive knot disease. *Pseudomonas savastanoi* and *Pantotea aggolomerans* produce and perceive the same acyl-homoserine lactone signals, C6-3-oxo-HSL (C63O) and C8-3oxo-HSL (C83O), which is an example of symmetrical sharing. On the other hand, *Pantotea agglomerans* uses two different signals, C6-HSL (C6) and C4-HSL (C4), one or both of which are perceived (“exploited”) by *P. savastanoi*. All three species produce indolacetic acid (IAA), which is a public good that causes the plant host mobilize nutrients for the bacteria (based on [27].).

**Figure 2:**
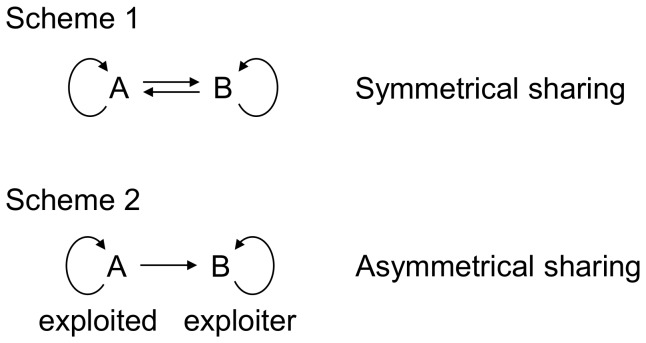
Scenarios for sharing signals and public goods in quorum sensing. Scheme 1: Symmetrical sharing. The two species, A and B, can both utilize the signals and public goods of the other species. Scheme 2: Asymmetrical sharing. Species B can utilize the signals and public goods of Species A, but not *vice versa*. The circular arrows indicate that each species is capable of utilizing its own signals and public goods.

Figure 1 illustrates two types of QS interactions. *PSV* and *ET* can mutually utilize the signals and public goods of each other, so this is a symmetrical relationship, which for two species, A and B, can be written as shown by Scheme 1, Figure 2. The arrows indicate that each species perceives its own signal as well as that of the other species. The other type of interaction is between PSV and PA. *PSV* exploits the signals of *PA*, while *PA* cannot utilize those of *PSV*. This is thus an asymmetrical relationship that can be written as shown by Scheme 2, Figure 2. The ternary bacterial consortium of the olive knot has another noteworthy feature - the production of the plant hormone indoleacetic acid (IAA) which is not regulated by QS [18]. IAA is essential for the tumorous growth of plant tissue which ultimately leads to knot formation [28]. Even though *PSV* is the *niche-maker* i.e. the only species within the trio that can infect the host alone, all three species contribute to knot formation by producing IAA which can thus be considered a public good within the consortium [27]. In the present work we try to answer the question of whether or not sharing signals and public goods in a symmetrical or asymmetrical fashion can *per se* contribute to the formation of stable bacterial communities.

Here we use agent-based *in silico* models to simulate the competition between QS bacteria that share signals, public goods and nutrients to varying extents. We show that bacterial species sharing public goods can easily form stable, co-localizing communities. We also show that relaxed specificity provides a fitness advantage for a bacterium when competing with other QS bacteria.

## Results

### Simulation Outcomes: Competition Phenotypes

Competition experiments were set up in such a way, that equal numbers of two species were placed randomly at the beginning of a longitudinal 2D surface “track” (see Methods). This track was covered with two kinds of nutrients (one for each species). At the beginning of the simulation, the cell agents were in the solitary (ground) state where they produced their own diffusible signals. As the simulation began, the cells started to move randomly, to feed, divide and continued to produce the diffusible signals. When the signal concentration in the environment reached a threshold, the cells switched to an active state, and started to produce their own type of public goods. When public goods in the environment reached a threshold concentration, the cells switched to the swarming state i.e. they increased their random movement, food intake as well as the production of signals and public goods. We used simulation conditions wherein a species needed to communicate and cooperate in order to reach the swarming state that enables the cell agents to survive. Viable species formed agent-communities that proceeded forward along the longitudinal track towards the nutrient. Such a viable community consisted of models in the swarming state, and it had a steady population size. When this population size was constant throughout at least 500 generations, the community was considered stable. Non-viable communities on the other hand remained stuck in the solitary state and could not move. Thus there was a clear difference between viable and non-viable communities.

**Figure 3:**
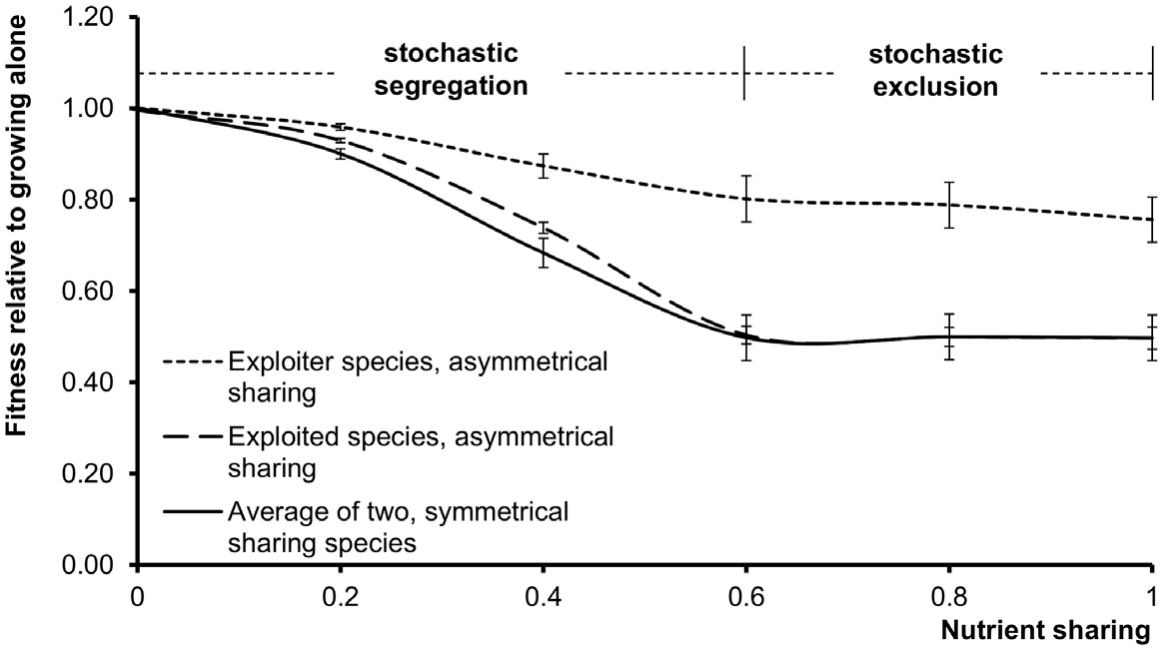
Competition outcomes observed with two competing QS agent populations (filled and non-filled circles). A) Stable, mixed community of two species (colocalization). Both types of cells are in the active, swarming state. B) Winning. The winner population forms a stable, swarming community (filled cells on top) while the loosing species (non-filled cells, near the starting position) will form a small community that will either stagnate in the solitary state, or die out, depending on the nutrients available. C1) Segregating populations. The species indicated with filled-dots is nearer to the resources, i.e. to the region of intact nutrients. C2) Patch-wise (mosaic-like) segregation. In the dfferent patches, either one or the other species is nearer to the resources.

In order to incorporate (symmetrical or asymmetrical) sharing into our model, we defined sharing coefficients for each species in such a way that zero value indicated no sharing and a value of 1.0 indicated complete sharing (See Methods for details). We defined different coefficients for signal sharing (a), public goods sharing (b), and nutrient sharing (c), respectively. It is noted that that the values of *a*, *b*, and *c* cover the entire *“competition space”*, i.e. *a* = *b* = *c = *0 denotes full independence of the competing species while, *a* = *b* = *c* = 1.0 denotes full sharing. Mapping out the competition space then consisted of carrying out experiments by varying *a*, *b* and *c* between 0.00 and 1.00 by steps of 0.02. Such an exercise requires a large number of simulations, each of them resulting in a final distribution of two bacterial agent populations which then has to be described in numerical and biological terms. In order to facilitate this task, we carried out preliminary experiments in order to explore the types of competition outcomes. Interestingly, we observed only a limited number of outcomes:

- *Co-localization, co-swarming*. In this case, the cells of the two species from a homogeneous mixed population (Figure 3A, Video S1) move together for at least 1000 generations. The segregation coefficient of this state is close to zero and the relative fitness of such a community could exceed 1.0, i.e. both constituent species can grow better in a community, than alone (eqn. 4, methods).
- *Winning*, *competitive exclusion*. Only one of the species could form a steady population (Figure 3B, top) while the other species, depending on the nutrients available, either died out or formed a small, stagnating population (Figure 3B, bottom, also see Video S2). By inspecting a large number of experiments conducted in a variety of conditions, we observed two types of winning scenarios. In one type, either of the two species could be the winner with a more-or-less equal probability. We termed this situation “*stochastic exclusion*”. In the other type of cases, the same species, i.e. the more competitive one, was always the winner - we termed this case “*competitive exclusion*”. The scenario of exclusion was apparently the same in both cases: the loosing species was left behind by the winner, with the distance growing between the populations. The loser species either started to stagnate at a very low population size or gradually disappeared). The relative fitness of the winner was 1.0 in all cases.
- *Segregation.* The two species formed two independent populations moving separately, one leading i.e. nearer to resources, and one lagging behind, i.e. farther from the resources (Figure 3C). In this case we observed two main types of outcomes. In one case, the same species, i.e. the more competitive one, was always nearer to the resources – we termed this case “*competitive segregation*” (Video S3). In the other case, either species could be the winner - we termed this case as “*stochastic segregation*”. In both cases, the *relative fitness I(fitness relative to growing alone)* of the leading species was 1.0 while that of the lagging one was lower, converging to zero. Interestingly, when we repeated the stochastic segregation experiments several times, in about half of the cases we observed “*patch-wise*” or “*mosaic-like segregation*” where one of the two separating species was leading at one location, while lagging at another one (Figure 3 C1, Video S4). In the leading patches (near to the resources), the relative fitness was close to 1.0 while in the lagging patches (farther from the resources) the relative fitness was lower. As a result, the average relative fitness of both species was lower than 1.0.

### Competition without QS

As a starting point, we carried out simulations with non-QS populations. As there are no signals and public goods in these systems, the growth rate of a species is solely determined by the nutrient intake.

**Figure 4:**
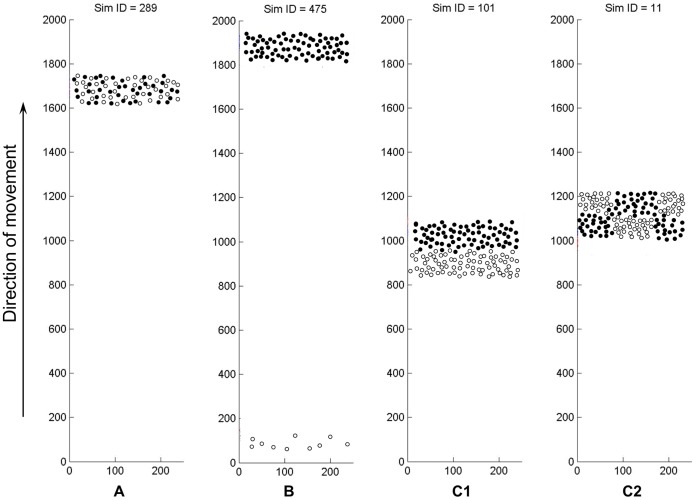
Competition of agent populations without QS. These systems lack signals and public goods, so the parameter space has only one variable, nutient sharing (denoted *c* in Methods). Relative fitness is defined in relation to the growth of the same species growing alone in the same conditions (eqn. 4, Methods). At lower nutrient sharing values the populations segregate. At higher nutrient sharing values, one of the populations goes extinct in less than 500 generations. When segregation and exclusion are stochastic, either species can be the winner or the loser with equal probabilities. Symmetrical sharing of nutrients (bottom curve) means that the two populations are equivalent, and their fitness decreases as nutrient sharing increases. Asymmetrical sharing of nutrients means that the exploiter species (top curve) can consume the nutrients of the exploited species (middle curve) but not *vice versa*. Note that the curve of the exploited species in asymmetrical sharing is virtually identical with the curve of the symmetrically sharing species. The values are the average of 10 calculations, error bars represent the standard deviation of the mean.

In the first competition experiments termed symmetrical sharing (Figure 4), each of the two species consumed its own nutrient, and in addition, it also consumed a part of the nutrient of the other species. This part was determined by the nutrient sharing coefficient *c* [0≤ *c* ≤1]. Nutrient sharing = 1.0 means that the two species consume identical nutrients, and in this case, we observed stochastic exclusion, i.e. either one or the other species died out with 50% probability. When the nutrients were not shared (nutrient sharing = 0), both species survived, and they segregated in a stochastic manner, i.e. either one or the other species was nearer to the resources. When nutrients were completely shared (*c* = 1.0), only one stochastically chosen species survived, so the relative fitness decreased to 0.5. In between the two extremes we saw a smooth transition, stochastic segregation was dominant at lower nutrient sharing, and stochastic exclusion was characteristic at higher sharing values, respectively.

In a second series of competition experiments termed asymmetrical sharing, each of the two species consumed its own nutrient, but only one of them, the “exploiter”, was able to consume the nutrient of other “exploited” species. In this case, the relative fitness of the two species was different (Figure 4, right), and they were equal only if the two species were independent in terms of nutrients (we note that in this case, there is no exploitation). Importantly, both segregation and exclusion were stochastic in nature.

It is worth mentioning that in both the symmetrical and asymmetrical cases, the relative fitness of a species never exceeded 1.0, i.e. the competition apparently always decreased the fitness of species as compared to the level of a species living alone in the same conditions. This is in fact expected, since the only interaction between the two species is competition for both resources (nutrients) and space.

Another important point was that the outcomes were stochastic in each case, i.e. either species could be the winner or the loser. According to Gause’s competitive exclusion principle [29], [30], if two species with different growth rates compete for the same nutrient, the fitter species will inevitably win. In our case, the two competing species are equally fit, so, by extension, one might expect a draw. However, the behavior of random-moving agents is known to be inherently stochastic, so the competition between agents is in fact expected to end with the victory of either one or the other agent species (stochastic exclusion). This is exactly what we see with our models without QS.

### Competition of Species with Symmetrically Overlapping QS Systems: Sharing

**Figure 5:**
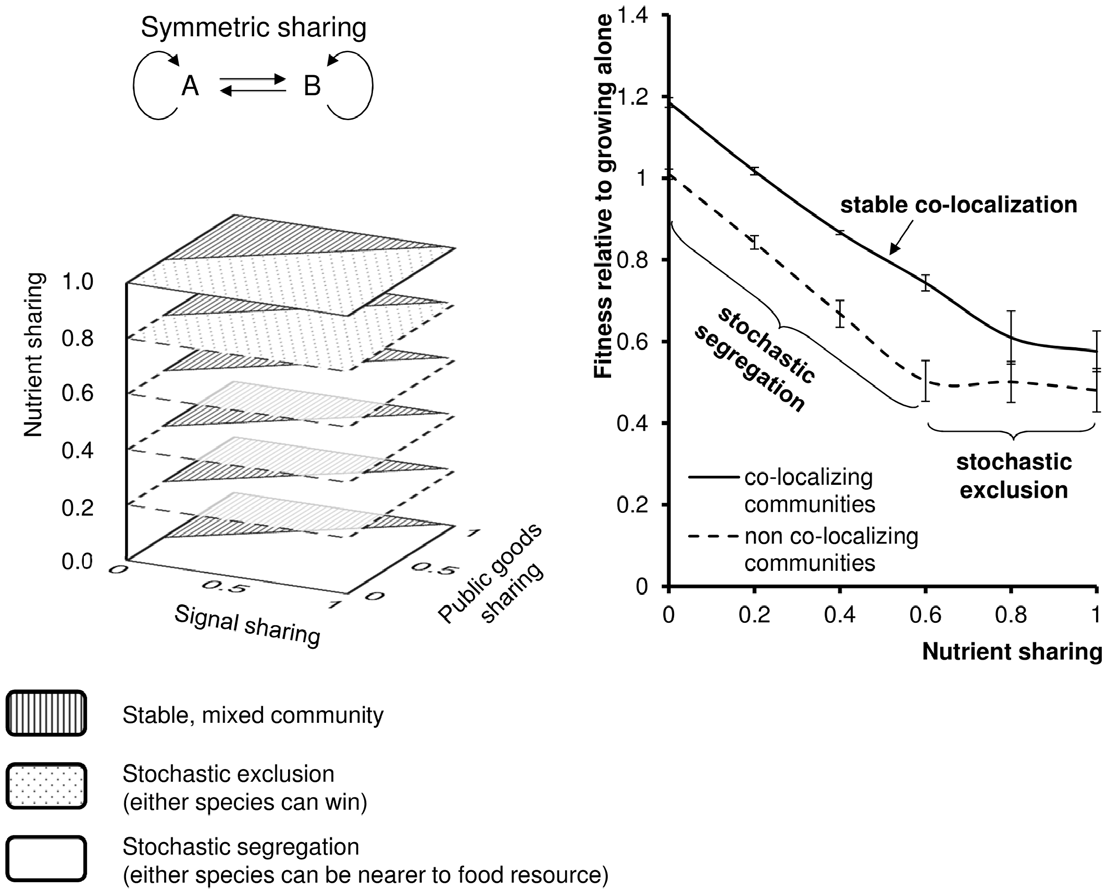
Sharing. Competition of species A and B that can utilize each other’s signals, public goods and nutrients to a varying extent. *a* = signal sharing, *b* = public goods sharing, *c* = nutrient sharing. Left: regions of co-colocalizing communities (i.e. segregation coefficient is below 0.5, see Methods). Right: Relative fitness of the mixed communities (shaded area on the left) as a function of food sharing (top curve). RF>1 indicates that both species grow faster in a community than alone. Bottom curve: relative fitness of non-colocalizing communities. The values are the average of 10 calculations, error bars represent the standard deviation of the mean.

In this scenario (Figure 2, Scheme 1) there is QS present, and both competing species can utilize the signals, public goods and nutrients of the other species (i.e. the interactions are symmetrical). The results of the simulations are summarized in Figure 5. The parameter space can be divided into two large compartments. In one of them (Figure 5A, shaded area) the two species can form stable, mixed (i.e. co-localizing) communities. This outcome was not observed when QS was not present. In addition, the relative fitness of both species was higher than 1.0 if nutrients consumed by the two species were at least partly different (Figure 5B, upper curve). This is a logical consequence, since two species can increase the performance of each other only if they mobilize independent resources for producing the common molecular signals and public goods. In the rest of the parameter space, the system showed a transition between stochastic segregation (at low nutrient sharing) and stochastic exclusion (high nutrient sharing). This behavior is thus identical to that seen with competition but without QS. As such we can conclude that symmetrical sharing of QS signals and public goods can lead to the formation of co-localizing, mixed communities if the public goods are shared. This happens in a substantial part of the parameter space, meaning that we can suppose that such mixed communities form relatively easily. Outside this region, QS apparently does not influence competition between the species.

### Competition of Species with Asymmetrically Overlapping QS Systems: Exploitation

**Figure 6:**
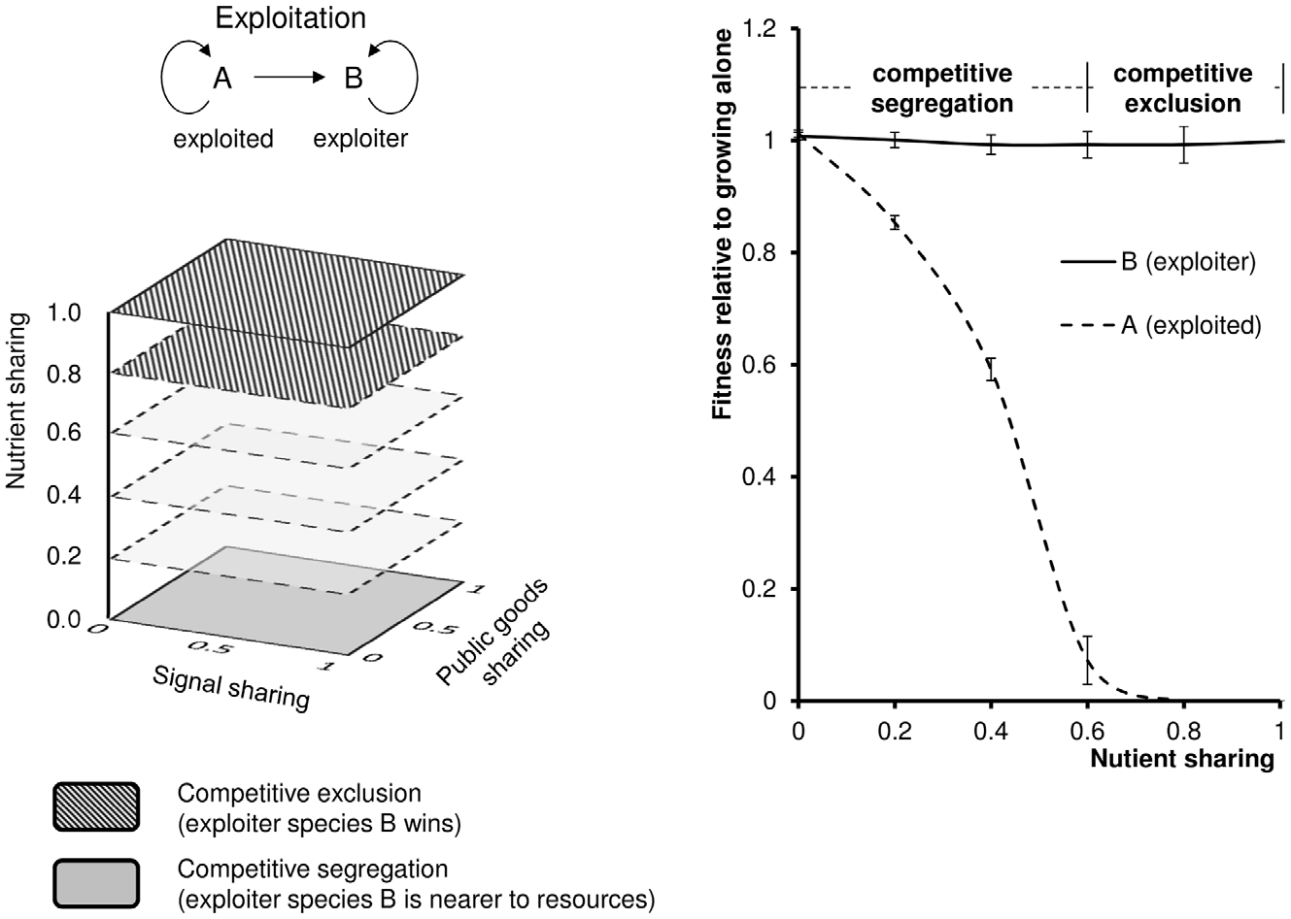
Exploitation. Species B exploits the QS system (signals, public goods) and nutrients of species A. This provides a fitness advantage to the exploiter species B in the entire parameter range. Left: Regions of the parameter space represent either competitive exclusion or competitive segregation. Right: Fitness of the two species relative to growing alone, as a function of nutrient sharing. Relative fitness = 1 in the top curve indicates that the growth of species B is not hampered by the competition. The values are the average of 10 calculations, error bars represent the standard deviation of the mean.

In this scenario (Figure 2, Scheme 2) there is QS present, both species are capable of surviving alone, but only one species (species B) can utilize the signals and public goods of the other species (species A). In other words, species B exploits the QS machinery of species A. The behavior of the system (Figure 6, left) was markedly different from the previous, symmetrical case. The difference was that the exploiter either clearly won, or, if segregated and stable populations form, it was always the exploiter nearer to the resources. In other words it seems that eavesdropping on the signals and/or parasitizing on the public goods of the other species clearly pays. In this case, the relative fitness of the two species are clearly different from each other (Figure 6, right), and they are equal only if the two species are independent in terms of signals, public goods and nutrients (we note that in this case, there is no exploitation) The differences between the two species are qualitatively shown on the plot of relative fitness vs. nutrient sharing.

The behavior of this system was qualitatively very similar to that of non-QS systems throughout the entire parameter space (Figure 3). The important difference was that here the exploiter has a unilateral fitness advantage even in the absence of nutrient exploitation.

## Discussion

In this work we sought to answer the question of whether or not sharing QS signals and public goods can influence the competition of two bacterial species. We carried out computational simulations of quorum sensing [31], [32] in which competing agent populations shared QS signals, public goods and nutrients to varying extents, and compared the simulation outcomes with those obtained without QS.

We found that mutual sharing of signals and public goods allows the formation of stable mixed communities in a substantial part of the parameter space (Figure 5). This outcome was not observed in the absence of QS. In the stable QS communities the members of the two competing agent populations were randomly mixed, i.e. the two populations were co-localized. As the two populations use increasingly different nutrients (*c* tends to 0), the co-operation produces a clear growth advantage as compared to both non-co-localizing species or to either of the two species grown alone (Figure 5, right). The use of different nutrients can be regarded as a case of metabolic complementarity, which has been experimentally observed in the case of several coexisting microbial consortia [33], [34]. On the other hand it is worth noting that forming a stable community may provide a fitness advantage for a variety of other reasons [23]. For instance, it was recently noted that two, co-swarming species can mutually help each other in situations where only one of the species is resistant to an antibiotic [32]. In other words, mixed populations can help the constituent species combine their skills, which is *per se* an advantage. A different tendency was apparent in the rest of the parameter space where the system tended to behave like those without QS. Namely, when the two species did not share nutrients, the result was stochastic, patch-wise distribution. Moreover, as nutrient sharing increased, the equilibrium shifted towards stochastic exclusion.

The analysis of symmetrical sharing suggested that sharing public goods and utilizing different nutrients is the key to forming co-localizing communities, while sharing QS signal seemed to be much less important – at least according to the present modeling scenarios. We think that this somewhat counterintuitive result follows from the fact that in our modeling experiments the two species were confined to the same space. On the other hand, it is known that external signals can recruit bacteria to precise locations via the well known mechanism of chemotaxis [35], and it was shown that agent models of QS bacteria are able track external signals [31]. This leads us to conclude that one of the plausible roles of shared signals is to attract bacterial species to each other via mutual chemotaxis so that they can act together if necessary.

We also found that unilateral exploitation of signals and public goods produced by one species provides a fitness advantage to the other, exploiting species within the entire parameter range (Figure 6). This situation is qualitatively very similar to non-QS competition. However we found that the exploiter of QS signals and public goods was a clear winner in all situations, in sharp contrast to the stochastic winning and loosing outcomes observed in the absence of QS. In other words, eavesdropping on QS signals and parasitizing on public goods is profitable. This finding suggests that cells equipped with a LuxR type receptor of broad specificity or harboring a LuxR solo [19] will have a fitness advantage because they will be able to respond to the signals of other competing species, which in turn may explain why receptors of relaxed specificity and LuxR solos are often observed in nature. A similar conclusion was reached by a recent article of Chandler and associates who studied the *in vitro* competition of two bacterial species that require QS for the production of antimicrobial factors that inhibit the adversary species [36]. In contrast, the present work suggests that the advantage of eavesdropping is not restricted to the special case of antimicrobial factors, i.e. the phenomenon seems to exist whenever signals and public goods are shared.

As this is an *in silico* study, a note on the scope and limitations of the modeling approach is appropriate. We use an agent-based approach with symbolic parameters that are not calibrated in terms of actually measurable physical quantities [31], [32]. As a consequence, the modeling results are qualitative i.e. they suggest only tendencies rather than exact values. The fact that the competitions described in this study led to biologically meaningful outcomes lends support to this approach. Second, the models assign identical metabolic efficiency (growth rate) to competing populations. This is not expected to occur in nature where interspecies differences are almost inevitable. At the molecular level, for instance, one cannot expect that LuxR proteins of two different species will produce a precisely identical effect in response to an AHL signal, and so on. In other words, species exactly equal in their fitness and their QS parameters are not likely to exist in nature. The meta-stable states found in our modeling experiments are also not likely to occur in nature. Consequently, we consider the meta-stable outcomes only as an indication of QS not influencing the competition at a given parameter combination.

In summary, we found that two factors, sharing of public goods and metabolic complementarity foster the formation of stable, co-localizing communities of QS bacteria. Sharing of signals was not found to sensitively influence the competitions, and so, based on earlier results [31], we suppose that the role of signal sharing is to help the different bacterial species to cluster at common locations. On the contrary, exploitation of the QS system of another species (eaves-dropping on signals and/or parasitizing on the public goods) tends to provide a unilateral fitness advantage to the exploiter, which may explain why promiscuous signal receptors and common presence of LuxR solos are observed in nature. In other words, our *in silico* study predicts that QS systems can fine-tune the equilibrium of bacterial populations.

## Methods

### Modeling

**Figure 7:**
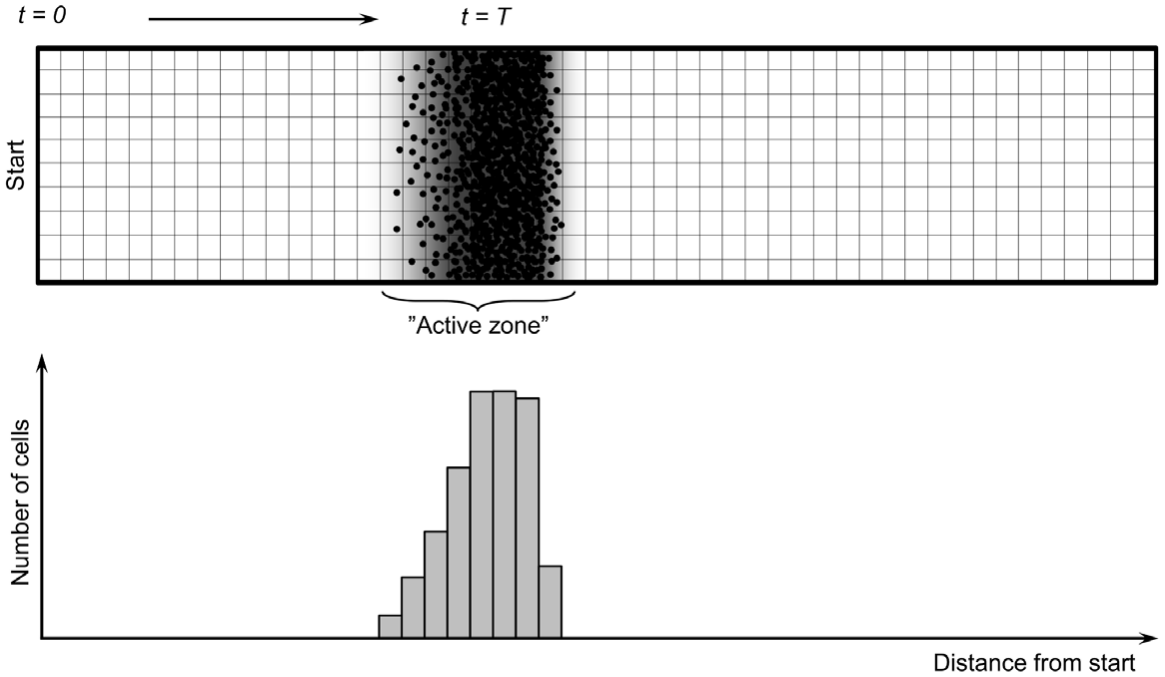
Principle of the dendrit growth model [**31**], [**32**]. The dendrite is modeled as a longitudinal, infinite 2D surface covered with a nutrient. Cell agents (black dots) placed at the start will begin to consume the nutrients and migrate. In the environment of the cell agents (the active zone) there are signals and public goods (indicated as grey area) sufficient to keep the cells in an activated state.

For modeling bacterial populations we used a model we previously developed [31], [32], with parameters summarized in Table S1. The model represents bacteria as random moving agents that move along a 2D longitudinal track corresponding to a dendrite of a colony growing on an agar plate. Cell agents release signals S and public goods F into the environment, while consuming a nutrient N evenly spread on the plate (Figure 7). When S reaches a threshold, cell agents enter an activated phase and increase their signal and public goods production. When public goods F reach a threshold, the cells enter a swarming phase with increased movement, S and F production. As a result, cell agents start to swarm (Video S5). In previous studies [31], [32] we have shown, that the model adequately describes the fundamental behavior of QS cells. We have shown, for example, that i) QS cells are able to follow external signals (Video S6); ii) Wild type QS cells form stable communities with cells that do not produce signals but can respond to it (Video S7); and iii) cheater cells that do not produce public goods will collapse a community of wild type cells (Video S8), even a very small number of cheaters can invade and collapse a healthy community (Video S9). In the competition experiments carried out in this work, we model two cell populations feeding on two kinds of nutrients (N1 and N2), producing and sensing two kinds of signals (S1 and S2) and two kinds of public goods, (F1 and F2), respectively.

We defined sharing coefficients for each diffusible material for each species. “a” and “b” determine the sensitivity towards the signal S and the public goods F of the other species, respectively, while “*c*” determines the fraction consumed from the nutrient N of the other species. The values of *a*, *b* and *c* are between zero and 1.0. For example, if Species1 understands only its own signal S1, the following equation will be used during the simulation:
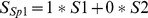
(1)


When Species1 consumes N1 and N2 in equal amounts, the following equation is used:
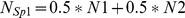
(2)


We will examine two different competition scenarios, namely symmetric sharing and exploitation (Details in the Results section). These two scenarios can be expressed by an appropriate choice of the multiplier coefficients, as shown in Table 1.

**Table 1:** Sharing coefficients for competing species used in the different scenarios.

	Signal			Public goods		Nutrients^1^	
	*S_1_*		*S_2_*	*F_1_*	*F_2_*	*N_1_*	*N_2_*
Scenario 1: Symmetric sharing							
Species A	1	*a*		1	b	(1−*c*)/2	c/2
Species B	*a*	1		*b*	1	*c*/2	(1−*c*)/2
Scenario 2: Exploitation							
Species A	1	0		1	0	1	0
Species B	*a*	1		*b*	1	*c*/2	(1−*c*)/2

1 Note that the multiplier of 1/2 in the definition of c follows from the condition of constant nutrient intake. This multiplier is not necessary in the case of signals (a) and public goods (b).

A modeling experiment within a given scenario (1–4 in Table 1) consisted of creating two competing populations, present in equal numbers (typically 1000 each), and letting them compete at given predetermined values of a, b and c, for 40,000 time steps. This corresponded to over 500 generations. The analysis of an entire scenario (e.g. symmetric sharing (Scenario 1, Table 1) consisted of varying the values of a, b, and c respectively between 0 and 1 in a grid-like fashion, with increments of 0.02. The data of the populations resulting after 40,000 time steps were stored after each simulation for numeric analysis and visualization.

### Numerical Characterization of Competing Populations

Agent populations were primarily characterized by their average size attained during the steady state of the simulation. The separation of two populations was calculated by an intuitive segregation index which was based on the work of Nadell et al. [37] and Mitri et al. [38]. This consisted of counting, for each agent, the members of its own population within an arbitrary number (in our case 10) of nearest neighbors, and calculating an average for the entire population. We scaled this measure in such a way that no overlap corresponded to 1.0 and a homogeneous mixture corresponded to 0.0 [39]. Note that the value of this index does not directly depend on how far the non-overlapping populations are from each other.

Fitness of a population was calculated as:
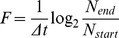
(3)where *F* is the fitness value, *Δt* denotes the elapsed time, and *N_end_* and *N_start_* are the size of the population at the beginning and end of the simulation respectively. Fitness is a dimensionless quantity that is often represented on a relative scale (dividing it by the fitness of a reference species) [37], [38]

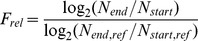
(4)where Frel is the relative fitness, and Nstart,ref and Nend,ref are the population sizes for the reference population. Note that the Δt terms are cancelled by the division. Our reference population was the same agent species growing alone (i.e. not in community with another species.). Therefore the Frel value calculated in this manner expresses the fitness difference caused by community formation. To make this distinction clearer, we term this quantity “fitness relative to growing alone”. The value of Frel is greater than 1.00 only if the community formation is beneficial for a species.

### Visualization of the Results

**Figure 8:**
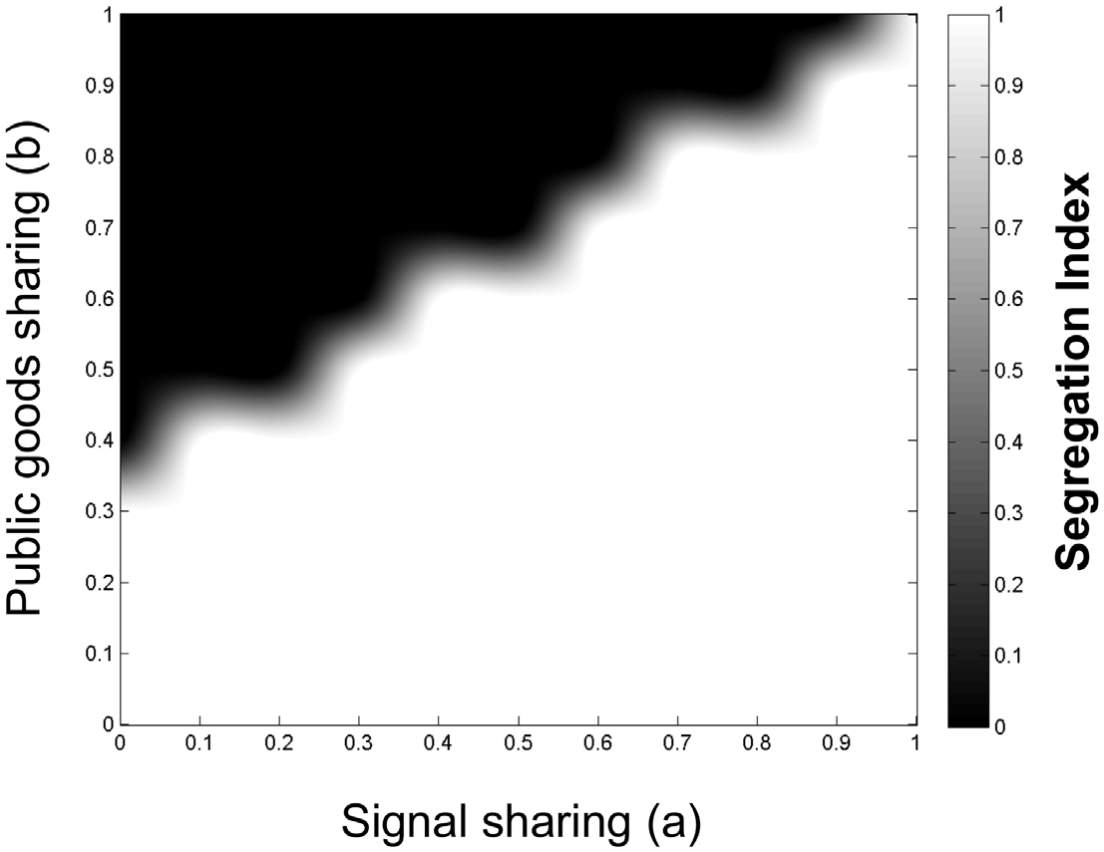
A heat map of segregation as a function of signal and public goods sharing. The black area indicates the parameter range wherein the two competing populations form a mixed community i.e. segregation coefficient is below 0.5. The data are from a simulation of asymmetrical sharing of signals and public goods at intermediate sharing of nutrients (*c* = 0.6).

As the simulations resulted in a great number of individual results, we used abbreviated forms of visualization of selected groups of simulations. Heat-maps were produced with public goods sharing versus signal sharing plots at given values of nutrient sharing. In a typical example (Figure 8), the parameter ranges were colored in a thresholded manner, i.e. different colors were assigned to areas that were above or below a threshold value of a separation coefficient or relative fitness. For the visualization of an entire scenario, such as asymmetrical sharing (Figure 6, left), graphically simplified heat maps were overlaid in 3D.

## Supporting Information

Table S1 **Parameters used for the simulations.**
(PDF)

Video S1 **Mixed community forming from two quorum sensing agent populations (partly) sharing signals and public goods and using (partly) different nutrients.** (symmetrical sharing, *a* = 0.3; *b* = 0.3; *c* = 0.1)
(AVI)

Video S2 **One quorum sensing population excluding the other species by competition.**
(AVI)

Video S3 **Two segregating quorum sensing agent populations (asymmetrical sharing,** ***a*** **= 0.3;** ***b*** **= 0.3;** ***c*** **= 0.1)**
(AVI)

Video S4 **Patchwise segregation of two quorum sensing agent populations (symmetrical sharing,** ***a*** **= 0.7;** ***b*** **= 0.2;** ***c*** **= 0.1)**
(AVI)

Video S5 **Swarming of a quorum sensing agent population.** As the simulation proceeds, the initial population grows to a much larger size, and this larger population proceeds at a constant swarming speed (steady state).
(AVI)

Video S6 **Tracking of an external signal by an agent population that does not produce the signal.**
(AVI)

Video S7 **Co-swarming of a wild type quorum sensing population (blue) with a population that does not produce the signal (green).** At the beginning the populations are present in equal quantities, as the simulation proceeds, a steady state is reached in which the population that does not produce the signal (green) is around 90%. The fluctuations of swarming speed and population size is more intensive in this steady state than in the case of a pure wild type community (Video S6).
(AVI)

Video S8 **Collapse of a wild type quorum sensing population (blue) by a cheat population that does not produce public goods (red).** At the beginning the populations are present in equal quantities. As the simulation proceeds, the non-cooperating population (red) first becomes the majority, then the community collapses and swarming stops.
(AVI)

Video S9 **Collapse of a wild type quorum sensing population (10 thousand cells, blue) by a small cheat population that does not produce public goods (10 cells, red).**
(AVI)
