# Correction: Stability of Multispecies Bacterial Communities: Signaling Networks May Stabilize Microbiomes

**DOI:** 10.1371/journal.pone.0111290

**Published:** 2014-10-10

**Authors:** The PLOS ONE Staff

The images for Figures 3 and 4 are incorrectly switched. The image that appears as Figure 3 should be Figure 4, and the image that appears as Figure 4 should be Figure 3. The figure legends appear in the correct order. Please see Figures 3 and 4 in the correct order below.

**Figure 3:**
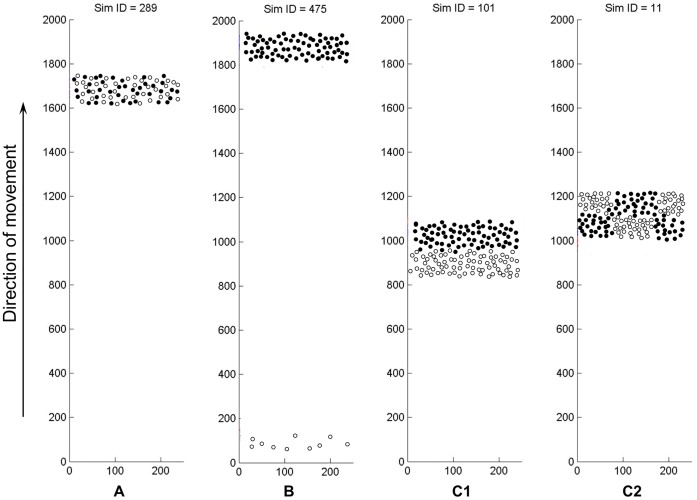
Competition outcomes observed with two competing QS agent populations (filled and non-filled circles). A) Stable, mixed community of two species (colocalization). Both types of cells are in the active, swarming state. B) Winning. The winner population forms a stable, swarming community (filled cells on top) while the loosing species (non-filled cells, near the starting position) will form a small community that will either stagnate in the solitary state, or die out, depending on the nutrients available. C1) Segregating populations. The species indicated with filled-dots is nearer to the resources, i.e. to the region of intact nutrients. C2) Patch-wise (mosaic-like) segregation. In the dfferent patches, either one or the other species is nearer to the resources.

**Figure 4:**
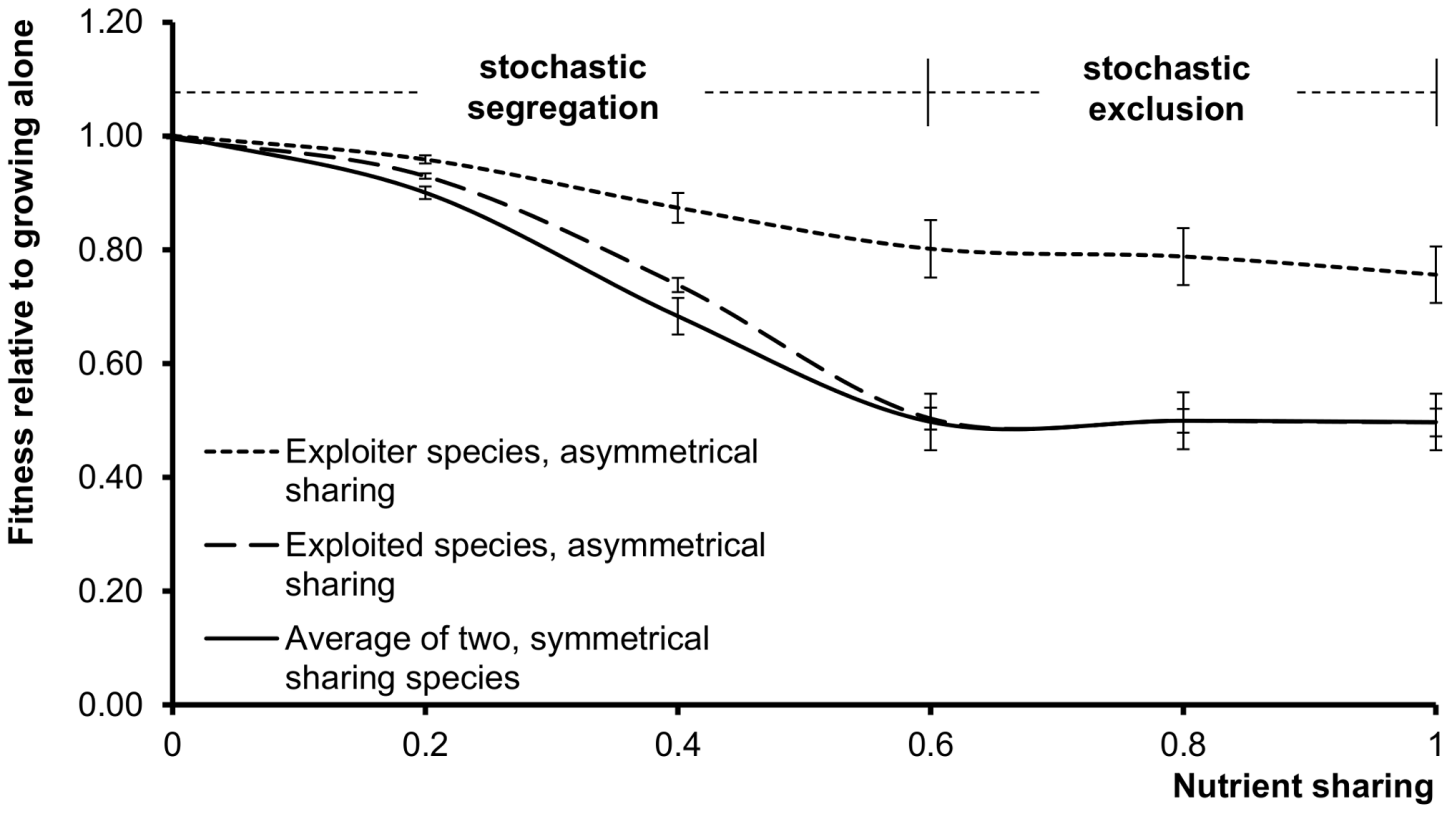
Competition of agent populations without QS. These systems lack signals and public goods, so the parameter space has only one variable, nutient sharing (denoted c in Methods). Relative fitness is defined in relation to the growth of the same species growing alone in the same conditions (eqn. 4, Methods). At lower nutrient sharing values the populations segregate. At higher nutrient sharing values, one of the populations goes extinct in less than 500 generations. When segregation and exclusion are stochastic, either species can be the winner or the loser with equal probabilities. Symmetrical sharing of nutrients (bottom curve) means that the two populations are equivalent, and their fitness decreases as nutrient sharing increases. Asymmetrical sharing of nutrients means that the exploiter species (top curve) can consume the nutrients of the exploited species (middle curve) but not vice versa. Note that the curve of the exploited species in asymmetrical sharing is virtually identical with the curve of the symmetrically sharing species. The values are the average of 10 calculations, error bars represent the standard deviation of the mean.
