# Supplementary material for: Stability of Multispecies Bacterial Communities: Signaling Networks May Stabilize Microbiomes
Source: PLoS One. 2013 Mar 4;8(3):e57947. doi: 10.1371/journal.pone.0057947 (PMC3587416; doi:10.1371/journal.pone.0057947)
Supplement: Table S1 — Parameters used for the simulations. (PDF) [file pone.0057947.s001.pdf]

Table S1. Parameters used for the simulations. The values are in arbitrary units.

Symbol	Description	Value(s)	Equation
n_{max}	Maximum simulation steps	40000	-
c_{max}	Maximum number of bacteria in cells	10	-
L_x	Size of racetrack (x dimension)	250	-
L_y	Size of racetrack (y dimension)	2000	-
L'_x	Size of the grid on racetrack (x dimension)	50	-
L'_y	Size of the grid on racetrack (y dimension)	200	-
C_N	Initial food concentration per cell	500	-
N_{start}	Initial number of bacteria	1000, 1000	-
v	Speed of bacteria (*solitary/ active/ swarming state*)	1.5 / 1.5 / 5.0	-
E_N	Energy from nutrient consumption	0.3 / 0.3 / 0.5	-
E_S	Energy spent on producing *Signal*	0.005 / 0.025 / 0.025	-
E_F	Energy spent on producing *Factor*	0.0 / 0.15 / 0.15	-
E_M	Energy spent on vegetative functions	0.1	-
E_t	Total stored energy for a bactium at *t* time	-	$E_{(t+1)} = E_t + E_N - E_S - E_F - E_M$
P_S	Amount of produced *Signal*	0.1 / 0.5 / 0.5	-
P_F	Amount of produced *Factor*	0.0 / 0.1 / 0.1	-
T_S	Threshold concentration of *Signal*	10	
T_F	Threshold concentration of *Factor*	10	
δ	Division threshold	12	
k	Constant for border advancement	0.5 / 0.1	$k = 0.05 / P_F$
BC	Border advancement counter	1	$BC_{(t+1)} = BC_t + (kF_{1,t}) + (kF_{2,t})$
D_N	Diffusion coefficient of nutrient	0.03	-
D_S	Diffusion coefficient of *Signal*	0.02	-
D_F	Diffusion coefficient of *Factor*	0.05	-
R_S	Decay constant of *Signal*	0.001	-
R_F	Decay constant of *Factor*	0.0001	-
F	Fitness	-	$F = \frac{1}{\Delta t}\log_2\frac{N_{end}}{N_{start}}$
F_{rel}	Relative fitness	-	$F_{rel} = \log_2\left(\frac{N_{end}}{N_{start}}\right) \Big/ \log_2\left(\frac{N_{end,wt}}{N_{start,wt}}\right)$
SG	Segregation Index	-	$SG = \frac{\sum_i \max(n_i)}{N_{pop}}$
SGN	Normalized Segregation Index	-	$SGN = \left(SG - \frac{1}{N_{species}}\right) \Big/ \left(1 - \frac{1}{N_{species}}\right)$
